# Bioinformatics for precision oncology

**DOI:** 10.1093/bib/bbx143

**Published:** 2017-12-18

**Authors:** Jochen Singer, Anja Irmisch, Hans-Joachim Ruscheweyh, Franziska Singer, Nora C Toussaint, Mitchell P Levesque, Daniel J Stekhoven, Niko Beerenwinkel

**Affiliations:** 1 Department of Biosystems Science and Engineering of ETH Zurich in Basel, Switzerland; 2 Department of Dermatology at the University of Zurich Hospital in Zurich, Switzerland; 3 ETH Zurich in Basel, Switzerland; 4 ETH Zurich in Zurich, Switzerland; 5 University of Zurich Hospital in Zurich, Switzerland

**Keywords:** cancer, molecular tumor board, data analysis pipeline, mutation calling, clinical decision support

## Abstract

Molecular profiling of tumor biopsies plays an increasingly important role not only in cancer research, but also in the clinical management of cancer patients. Multi-omics approaches hold the promise of improving diagnostics, prognostics and personalized treatment. To deliver on this promise of precision oncology, appropriate bioinformatics methods for managing, integrating and analyzing large and complex data are necessary. Here, we discuss the specific requirements of bioinformatics methods and software that arise in the setting of clinical oncology, owing to a stricter regulatory environment and the need for rapid, highly reproducible and robust procedures. We describe the workflow of a molecular tumor board and the specific bioinformatics support that it requires, from the primary analysis of raw molecular profiling data to the automatic generation of a clinical report and its delivery to decision-making clinical oncologists. Such workflows have to various degrees been implemented in many clinical trials, as well as in molecular tumor boards at specialized cancer centers and university hospitals worldwide. We review these and more recent efforts to include other high-dimensional multi-omics patient profiles into the tumor board, as well as the state of clinical decision support software to translate molecular findings into treatment recommendations.

## Introduction

The continuous improvement, greater availability and decreasing cost of next-generation sequencing (NGS) have allowed major cancer centers worldwide to offer NGS-based personalized oncology for clinical practice. The goal is to profile the genetic aberrations of tumors such as single-nucleotide variants (SNVs), copy number variants (CNVs), insertions and deletions (indels), structural variants (SVs) and gene fusions, and to suggest potential treatments based on the molecular lesions that are observed. These approaches can be organized either as a single institutional molecular tumor board (MTB), where detected genetic aberrations will be evaluated for any potential matching treatments, or as a basket trial, in which predefined genetic alterations are assigned to matching treatment arms (baskets). Both approaches typically include patients who are progressive on all conventional treatment options and those with rare cancers for which limited treatments exist, such as many pediatric tumors [1].

**Figure 1:**
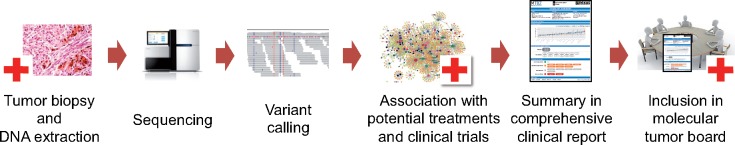
Schematic overview of the workflow of a MTB. Tumor biopsies are obtained from consenting patients, and DNA is extracted and sequenced. Variants are called and then annotated and prioritized for potential functional or clinical relevance before being reported to a tumor board, where an interdisciplinary team decides about treatment options.

MTBs are now widespread in the USA, Europe and Australia with reported patient numbers to date ranging up to 2000 patients per cancer center [2]. Ideally, a biopsy is taken on tumor progression from the last therapy to resemble the current genetic state of the evolved tumor [3, 4]. However, some MTB approaches also profile biopsies sampled at diagnosis, especially for high-risk tumors with few treatment options [5–7], or biopsies of patients currently responding to therapy but without further therapeutic options [8–10]. Typically, biopsies with a tumor content of at least 20% are analyzed by cancer-specific gene panels, such as FoundationOne [11], or whole-exome sequencing (WES) [12] (Figure 1). Some centers include additional measurements, such as profiling of the transcriptome, methylome or copy number alterations [5, 13, 14]. Whereas profiling by WES usually includes a germ line control [5, 12], this control is missing in most panel sequencing approaches [2, 11]. In an ideal setup, matched tumor–normal DNA and RNA sequencing samples are processed in the same conditions, including in the same lane of the sequencer. The resulting NGS data are analyzed for genetic aberrations and potential drug interactions. Specific treatment suggestions are, after careful consideration of available preclinical and clinical evidence, incorporated into a clinical report, which together with the patient’s clinical data, such as treatment history, comorbidities and radiology scans, forms the basis for therapeutic decision-making in an interdisciplinary MTB. The molecular report may suggest tumor genotype-matched clinical trials and targeted therapies, such as kinase inhibitors, or recommend the avoidance of drugs, for example, in cases where mutations that potentially confer treatment resistance have been detected.

A number of challenges exist for current precision oncology approaches during all the steps of the process, starting from clinical sampling up to bioinformatics analysis, reporting and patient treatment. In addition to difficulties in obtaining a tumor biopsy and a sufficient quantity and quality of tumor DNA and RNA for molecular profiling, Massard *et al.* [14] reported that in less than half of 843 patients with advanced solid tumors, an actionable mutation was found. In the largest basket trial approach to date, the MATCH trial of the US National Cancer Institute (NCI), the restricted number of drug arms resulted in even fewer gene–drug matches. Only 9% of the patients could be assigned to a genetics-based treatment [15]. The development of more selective drugs over time is expected to increase these numbers. A further challenge is to translate a MTB suggestion into patient treatment. Beltran *et al.* [12] reported that although 94% of solid cancer patients in their MTB had an actionable alteration, only 5% were treated based on their genotype. The main reasons were rapid decline of condition and, more importantly, the lack of access to clinical trials or off-label drugs. Finally, the costs of molecular profiling can be challenging as well. Although it has been shown that panel sequencing is financially feasible [16], the costs of more comprehensive approaches such as WES, whole-genome sequencing (WGS) and RNA sequencing can be prohibitive for reimbursement. Nevertheless, it is to be expected that comprehensive sequencing will become cheaper, and therefore financially feasible.

The final outcome of cancer genotype-matched patient treatment, namely, patient response to treatment, varies widely in the published literature. Schwaederle *et al.* [11] report a partial response in 36% of patients, whereas the MOSCATO trial [14] reports objective responses in 11% of patients receiving matched treatments. The currently ongoing basket trials such as NCI MATCH, which aims to include 6000 patients, will provide more conclusive data, owing to larger cohort sizes and well-defined genotype-matched treatment arms. Nonetheless, a single-gene aberration is not always predictive of treatment response as has been observed for the oncogenic BRAF mutations, which predict BRAF inhibitor response in melanoma [17], but not necessarily in non-melanoma cancers [18]. Furthermore, molecular tumor approaches reported to date are based on profiling of single biopsies. Large-scale sequencing studies have shown extensive intra-patient heterogeneity between different metastases and even within individual tumors [3, 4], indicating that this approach might not necessarily identify ubiquitous and also miss relevant alterations.

In this review, we discuss bioinformatics approaches to NGS-based precision oncology, including variant calling, annotation, interpretation, drug matching and reporting in a MTB setting. We have set up a bioinformatics analysis pipeline and reporting workflow for WES and WGS at the MTB of the University Hospital Zurich and will base this review on our experiences with this ongoing effort. For guidelines on the analysis of NGS-based oncology panels, please refer to [19].

## Requirements on bioinformatics solutions for clinical oncology

High-throughput NGS allows for time- and cost-effective molecular probing of tumors. However, the resulting sequencing data is challenging to analyze because of its large size and various confounding sources of variation, most notably amplification and sequencing errors. Careful analysis of NGS data is particularly important in the context of MTBs, where treatment suggestions based on mutation calls may have dramatic effects, ranging from recovery to death of a patient. Therefore, strict standards with respect to several aspects described below need to be followed.

First and foremost, experimental noise needs to be distinguished from true biological signals. Treatment decisions have to be based only on validated, real biological alterations and should not be misled by technical artifacts. Toward this end, appropriate computational data analysis pipelines have to be used that cover the entire process from primary analysis of the read data to clinical reporting. To understand the limitations of an implemented pipeline, it needs to be evaluated under defined conditions reflecting realistic use case conditions [20, 21]. Pipelines need to be robust with respect to new sequencing data that may differ in some aspects from previously analyzed samples. In addition, mutation calls should be reported with a confidence estimate. Although some mutation callers report, for example, *P*-values or posterior probabilities, it remains a major challenge to provide a meaningful notion of confidence for the results of an entire pipeline. This is particularly important, as the overlap of different approaches is often limited, as mentioned in [22–25].

The results produced by a bioinformatics pipeline have to be reproducible. This requirement entails several technical prerequisites discussed below and includes controlling random seeds for all steps that involve randomization. Another important aspect of reproducibility is a rigorous documentation of each step of the pipeline, including complete documentation of the used tools, their version and parameter settings. This also holds for databases and ensures complete transparency [20]. For instance, in the past, most genomic studies have used as a reference genome *GRCh37* from the Genome Reference Consortium or its equivalent from the University of California Santa Cruz, version *hg19*. Even though there are only minor differences in their genetic information, the naming scheme is different, which can lead to confusion. Moreover, the new human genome assembly *GRCh38* not only updated the main chromosomes, and therefore changed their coordinates, but also included new contigs to represent population haplotypes, further complicating reproducibility. Therefore, it is necessary that for each file used in the pipeline, its generation and dependencies are clearly described. Such a setup also guarantees the traceability of all results. For example, it should be possible to trace back the call of a treatment-critical mutation, to assess the call manually and to validate it before recommending the treatment. In addition, genomic alterations in the patient which are not directly linked to cancer, known as incidental variants, may be discovered. As these variants may be reported in various ways with potential ethical implications, a clear strategy needs to be defined, for example, reporting all relevant incidental findings [26].

In addition to these requirements on stability, robustness, reproducibility and traceability of the computational pipeline, the size, sensitivity and complexity of comprehensive clinical data sets combined with the urgency caused by the often critical state of the respective patient result in a set of challenging technical prerequisites for the computational infrastructure and the implemented data analysis software of an MTB.

## Technical prerequisites

Medical data require secure data storage and distributed computing. Secure storage of sensitive data calls for restrictive authorization and authentication schemes that limit data access to those who hold valid credentials. These schemes have to be implemented and reviewed on a regular basis, in particular in a clinical setting in which data might have to be stored for many years. As data sets grow and the analysis becomes increasingly complex, the computation time of even single data sets outgrow the capacity of individual computers. Distributed computing, such as high-performance clusters or cloud engines, allows for efficient execution of data analysis workflows. The drawback is that these instances do not natively comply with the strict security requirements of medical data, as resources are shared among users with and without sufficient permissions.

To address the strong requirement for speed, accuracy and reproducibility, the use of a workflow manager can help with standardization and automation of the analysis. Multiple workflow managers are available such as Snakemake [27], Nextflow [28], Toil [29], Bpipe [30] and to some extent also the Galaxy framework [31]. Although they differ in features such as cluster support and programming language, they have all been implemented with the same rationale: the scientist defines the order, the parameters and the input data for a chain of tools, and the workflow manager takes care of the correct execution and documentation of the intermediate steps.

## Primary analysis of DNA data

**Figure 2:**
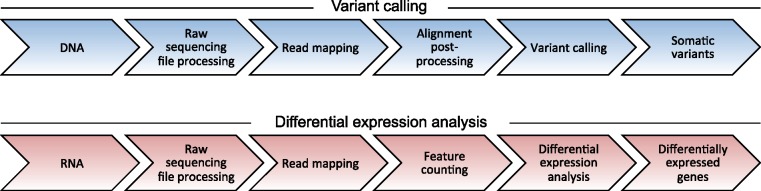
Schematic overview of analysis steps for DNA variant calling (blue, top) and RNA expression analysis (red, bottom).

The primary analysis of genomic data sets typically starts with the raw sequencing data and finishes with a list of mutations. The different steps of this analysis are conducted in complex pipelines that differ according to the sequencing method used. Even for the same type of sequencing method, many pipelines are available and it has been observed repeatedly that the results can be different [24, 25, 32–35]. The primary analysis can be subdivided into (i) raw sequencing file processing, (ii) read mapping, (iii) alignment post-processing and (iv) variant calling (Figure 2). These steps are implemented to different extents in most pipelines. In the following, we will describe each of them briefly.

### Raw sequencing file processing

The genomic sequencing data are provided in the form of reads, amplified DNA sequences of tens to hundreds of base pairs, in so-called FASTQ files. In addition to the sequencing information, for each nucleotide, the FASTQ file contains quality scores provided by the sequencing machine. These quantities represent the probability of the reported nucleotide to be a sequencing error, as estimated by the sequencer. Quality scores can be used to trim reads such that the FASTQ files only contain high-confidence nucleotides, and the number of false positive calls owing to sequencing errors is kept at a minimum [36]. Another source of artifacts are sequencing adapters. Adapters are short nucleotide sequences attached to the genomic DNA fragment and used for amplification and sequencing. Sometimes these adapters are contained within the nucleotide sequence of a read and may lead to false-positive mutation calls. Therefore, many pipelines include tools such as Cutadapt [37], Trimmomatic [38], SeqPurge [39] or Flexbar [40] to remove low-quality bases and artifacts in the raw sequencing data.

### Read mapping

Owing to the sequencing protocols, the reads do not contain any information about their origin in the genome. This information is inferred by using read mappers, which align, or map, all reads to a given reference sequence. The importance of this time-critical step has led to the development of >60 different read mappers [41], with BWA [42] and Bowtie2 [43] being popular examples. They usually provide their results in Sequence Alignment/Mapping format (SAM, binary version BAM) files, which undergo different modifications during the alignment post-processing step.

### Alignment post-processing

This phase typically starts with sorting the SAM/BAM files according to their genomic coordinates. Afterward polymerase chain reaction (PCR) duplicates are often removed using, for example, picard tools (http://broadinstitute.github.io/picard) or SAM tools [44]. These duplicates are copies of the same genomic fragment and indicate selective PCR amplification which can bias the analysis. However, duplicated reads can also be biological copies originating from the same genomic location of chromosomes of different cells. The probability of a duplicate read to be a biological copy increases with coverage [45], such that this step is typically not performed for deep-coverage targeted sequencing approaches.

Another post-processing step is the re-alignment of reads around indels. As read mappers rely on heuristics to deal with the large amount of data, the resulting alignments can be suboptimal. This is especially true for sites harboring indels because here the difference between the reference genome and the patient reads is more pronounced. To reduce this bias, many pipelines perform re-alignments around these positions, for example, using the Genome Analysis Toolkit (GATK) [46–48]. For Illumina data, GATK also provides a tool to correct for biases in the sequencing process, which uses a machine learning approach to re-compute the quality scores of the nucleotides. The use of the re-alignment and quality score recalibration is generally recommended [47, 49], but they are not always performed in practice, as they are time-intensive and the impact is sometimes not obvious [50, 51].

### Variant calling

Variant calling in the context of oncology refers to the identification of somatic variants in the cancer genome. These variants have occurred during the development of the tumor and they need to be separated from germ line variants of the patient. Targeted cancer therapy aims to selectively inhibit cells with specific somatic mutations, such as SNVs, indels and SVs. There are two conceptually different approaches to identify somatic variants, namely, (i) filtering for somatic variants using existing variant databases and (ii) using a normal control sample to distinguish somatic from germ line variants.

The first approach identifies variants in the genome by analyzing only the tumor sample, using tools such as VarScan2 [52], SiNVICT [53] or GATK HaplotypeCaller [46–48]. The identified mutations are then compared with existing databases, such as dbSNP [54, 55], ExAC [56], ClinVar [57] or COSMIC [58], to assess whether a given variant has previously been reported as a germ line variant or a cancer-associated change in the genome. The major advantage of such approaches is independence from a control tissue sample, while major drawbacks are dependence on quality and completeness of the databases as well as limited sensitivity because low-frequency variants are difficult to distinguish from sequencing noise.

The second approach uses an additional non-cancerous sample from the same individual as a germ line control. This approach can further be subdivided into methods that (a) apply variant calling to the tumor and control sample independently (using tools of approach (i)) or (b) use the genomic information of the two samples jointly. Approaches in the first category subtract from the tumor sample all mutations in the control sample, i.e. the germ line variants. Methods of the second category directly call somatic mutations by comparing variants between tumor and control sample for each position, which increases the power for calling true mutations at a given false-positive rate [59]. The idea is to model the control and tumor sample jointly to transfer noise patterns learned from the control sample to handle confounding factors appropriately. The results of approaches in (b) are usually superior to results from approaches in category (a), especially with regard to specificity [60]. Examples are MuTect [61], Strelka [62], VarScan2 [52], JointSNVMix [60] and deepSNV [59, 63].

For the identification of SVs, there are four commonly used techniques, namely, clustering, split-read mapping, contig assembly and statistical testing, as described in more detail in [22]. SV detection can be divided into CNV detection and identification of other SVs such as translocations and inversions. CNV calling is performed not only on WGS, but also on WES and even amplicon sequencing data. Numerous methods for CNV calling exist [64], including EXCAVATOR [65], BIC-seq2 [66] and CopywriteR [67]. In contrast, SVs like translocations and inversions are usually called based on WGS to determine the actual breakpoints of the genomic rearrangement. Popular methods include Pindel [68], SVDetect [69], Delly [70] and Lumpy [71]. As mentioned in [22], sensitive and specific SV calling remains a challenge, and choosing the appropriate approach greatly depends on the type of SV and NGS protocol features, such as the library size. For a more comprehensive review of CNV and SV calling, we refer to [22, 64, 72, 73].

## Primary analysis of RNA data

While variant calling is typically based on DNA data, differential expression analysis uses RNA sequencing data. Alignment and read pre- and post-processing are generally similar for DNA and RNA sequencing, with some key differences, for example, read mappers have to perform a special gapped alignment, because RNA reads sometimes do not continuously align to the reference sequence owing to splicing events, but map to different exons with large gaps in between. Popular RNA aligners are STAR [74] and TopHat [75].

In contrast to DNA alignments, the coverage of RNA alignments varies between regions in the genome owing to different gene expression levels. Thus, the coverage of RNA alignments can be used to infer gene expression levels after normalization with respect to total read count, gene length and possibly other confounding factors such as GC content. Here, commonly used tools include HTSeq [76] and featureCounts [77].

If matching control tissue is available, differential gene expression compared with normal can also be assessed, albeit with reduced statistical power owing to the lack of replicates. Typically, however, no adequate normal tissue is available. Popular tools for differential gene expression analysis include DESeq2 [78] and EdgeR [79, 80], which model read counts directly, account for various sources of confounding and provide robust statistical procedures for parameter estimation.

An alternative, albeit imperfect, approach to detecting over- or under-expressed genes is the comparison of tumor gene expression levels to publicly available data sets of suitable tumor or normal cohorts, such as TCGA (https://cancergenome.nih.gov/) or GTEx [81]. For example, Oberg *et al.* used 124 transcriptomes from various normal tissues as a reference data set in a pediatric hematology-oncology setting [5]. Batch effects have to be taken into account, when comparing separately generated RNA sequencing data sets. Multiple tools for batch effect removal are available, e.g. the R package SVA [82].

However, it remains a challenge to integrate transcriptome data in a clinical tumor board setting, where the task typically is to compare an individual tumor sample with a separate healthy reference or tumor cohort. Eventually, the goal is to use the RNA sequencing data in at least three ways: (1) to validate the expression of SNVs, CNVs or SVs, (2) to identify misregulated pathways that could potentially be targetable and (3) to determine the proportion of immune cell infiltration based on immune signatures. For each of these aims, different references might be necessary. As healthy tissue from individual cancer patients is not always available, public transcriptome databases may be used as a comparison. However, the transcriptional changes between healthy controls and cancer cells may be less revealing than a comparison with similar cohorts of cancer biopsies. For instance, different subtypes of melanoma (i.e. mucosal versus uveal or cutaneous) have some similarities, but differences might reveal informative vulnerabilities that could be targeted in a MTB setting. Lastly, the ability to infer tumor infiltration of immune cells based on RNA expression could be a powerful means to complement traditional immunohistochemistry approaches that are still relevant for predicting response to immunotherapies.

## Variant annotation

The process of variant annotation aims at assembling as much relevant information as necessary to select or discard a given variant while at the same time keeping the amount of information that needs to be parsed manually as small as possible. Possible annotations range from basic attributes like affected gene, coding or noncoding, synonymous or nonsynonymous to complex classifications like clinical significance.

Clinical significance is the most relevant piece of information for a clinician about any variant. Typically, variants are categorized as pathogenic, likely pathogenic, of unknown significance, likely benign, or benign. However, the classification of specific variants is not consistent across available databases such as ClinVar [57], CIViC [83], COSMIC [58] and dbSNP [54, 55]. For instance, algorithms such as SnpEff [84] categorize variants based on the predicted impact on protein function, whereas ClinVar [57] links particular variants to known functional or clinical features.

Additionally, the vast majority of detected variants have not yet been assigned a level of functional relevance or clinical significance. Thus, focussing only on variants annotated as (likely) pathogenic will often result in no variants at all being reported. This is unsatisfactory and potentially misleading. Annotation tools such as SnpEff [84] and ANNOVAR [85] can be applied to help extract interesting variants for the clinical report. Furthermore, a useful database for the identification of potentially deleterious SNVs is dbNSFP [86]. It contains predictions from a large set of functional prediction tools for all possible nonsynonymous SNVs and splice variants in the human genome. Among others, annotations include deleteriousness and affected protein domains. Both can be very useful for variant prioritization. For example, a deleterious variant ranks higher than a non-deleterious variant and a nonsynonymous coding variant within a protein domain ranks higher than a non-protein-truncating variant outside of a protein domain. For functional effect prediction of indels, PROVEAN [87] can be used. It predicts the functional effects of single and also multiple amino acid substitutions, in-frame insertions and deletions.

Another helpful annotation when it comes to variant prioritization is whether a variant affects a potential cancer driver gene. Information on genes that have been reported as driver genes can be obtained from the literature [88, 89] and databases such as UniProt [90], IntOGen [91, 92] and COSMIC [58].

With the goal of recommending drugs, it is useful to annotate genes with drugs that target them. Popular online resources to query drug–gene interactions are DGIdb [93, 94], OncoKB [95] or CIViC [83]. It would be desirable to also annotate genes with indirectly interacting drugs, i.e. drugs that target proteins up- or downstream of the gene within the relevant pathway. Such annotation methods are currently being developed, e.g. [96], but no easy-to-use tool or API has yet been established.

## Interpretation of molecular profiles and clinical reporting

Interpreting the clinical significance of genomic variants and transcriptional changes, i.e. the synthesis of all available information about an event and its relevance to clinical action [97] is a daunting and laborious task. It constitutes the bottleneck of the whole process from biopsy collection to reporting to the MTB [97] because it cannot be fully automated in a reliable way. Nevertheless, a properly curated list of evidence-based therapy recommendations forms the basis for the MTB to decide on the treatment of a patient. Thus, the ultimate goal of clinical reporting is to apply clinical interpretation to select relevant variants and to recommend targeted, personalized therapies [98].

The best case scenario for reporting is a single pathogenic mutation with an associated, clearly defined and clinically verified therapy, such as BRAF^V600E^ and vemurafenib [17]. However, more often, several damaging mutations of unknown significance are identified and it is unclear which, if any, have functional or clinical relevance. This is especially true in the case of comprehensive sequencing. Consequently, the potentially long list of mutations and their associated drugs needs to be filtered automatically to obtain a relevant but manageable selection of drug–gene interactions that can then be further curated manually. Examples of such filters are exclusion of non-cancer drugs or of drugs with a nonsensical mode of action for their associated mutation, such as an inhibitor for a deleted gene.

For the report, each listed drug–gene association has to be assigned a level of confidence. In 2017, the Association for Molecular Pathology, the American College of Medical Genetics and Genomics, the American Society of Clinical Oncology and the College of American Pathologists have established four evidence levels based on professional guidelines as well as size and number of studies supporting a mutation and its associated drug [99]. While these categories may or may not fit to the local or national situation of a reporting facility, the adherence to a joint consensus is favorable, as it facilitates the comparison with other resources, like OncoKB [95] and PharmGKB [100], and also the longitudinal use of findings in the clinic.

As mentioned above, it is not unusual for variants to be assigned contradicting levels of clinical significance across and even within individual databases. Therefore, preparation of a meaningful tumor board report often needs to include a manual investigation of the associated literature to properly annotate and clinically interpret the identified variant. To determine the clinical actionability of a variant, one can consider, for example, the cell type content of the biopsy, the tissue-specificity of gene expression alterations and, when not using germ line controls, potential germ line variants. Alternatively, all findings, even contradictory ones, can be reported, thereby leaving the entire interpretation up to the MTB. However, it is questionable whether the latter approach is a practical solution given the often very short time frame that is available in the MTB to discuss particular cases. This trade-off between comprehensiveness and conciseness is a common theme in clinical reporting.

## Molecular Tumor Board Zurich

In early 2015, we started the Molecular Tumor Board Zurich (MTBZ) to comprehensively profile and report on end-of-treatment line melanoma patients [101]. An important prerequisite for the success of this endeavor was to bridge the gap between the medical and technical disciplines and establish a common language to better understand the needs for efficient and effective reporting to the tumor board.

The goal of this project was to overcome certain shortcomings in the standard of care. We address these issues by (i) comprehensive sequencing, (ii) automated and comprehensive annotation, (iii) investigation beyond disease-specific therapies and (iv) identification of therapies with lacking or reduced efficacy. For patients without any traditional treatment options remaining, comprehensive profiling of the tumor might offer new treatment options. Therefore, we established a protocol based on WES and WGS of tumor and matched normal samples, specifically WES for SNV and small indel calling and low-pass WGS for CNV calling. In addition to the identification of somatic variants, WES allows us to provide more information potentially relevant to the clinician, namely, mutational burden and the patient’s HLA type. We report the mutational burden of a tumor, which is especially useful for the decision on using immunotherapies, for instance, in the case of CTLA-4 blockade in melanoma [102]. Further, we put it into context by comparing it with the distribution of mutational load within publicly available samples from the same and other cancer types [103]. The HLA-I type of a patient, which can be inferred from WES data using, for example, OptiType [104], provides information on eligibility for certain cancer vaccination trials [105]. Another important difference to standard procedures is the implementation of an automated and comprehensive annotation pipeline querying multiple databases for clinical significance, finding clinical trial opportunities worldwide and putting observed variants into the context of large studies like TCGA using the cBio Cancer Genomics Portal [106]. The use of the latter is twofold: We can assess (i) whether a variant is typical for the cancer type which improves confidence, and (ii) whether a variant uncommon in the given type of cancer is commonly observed in another type of cancer and could explain why previous standard treatments had not been successful.

**Figure 3:**
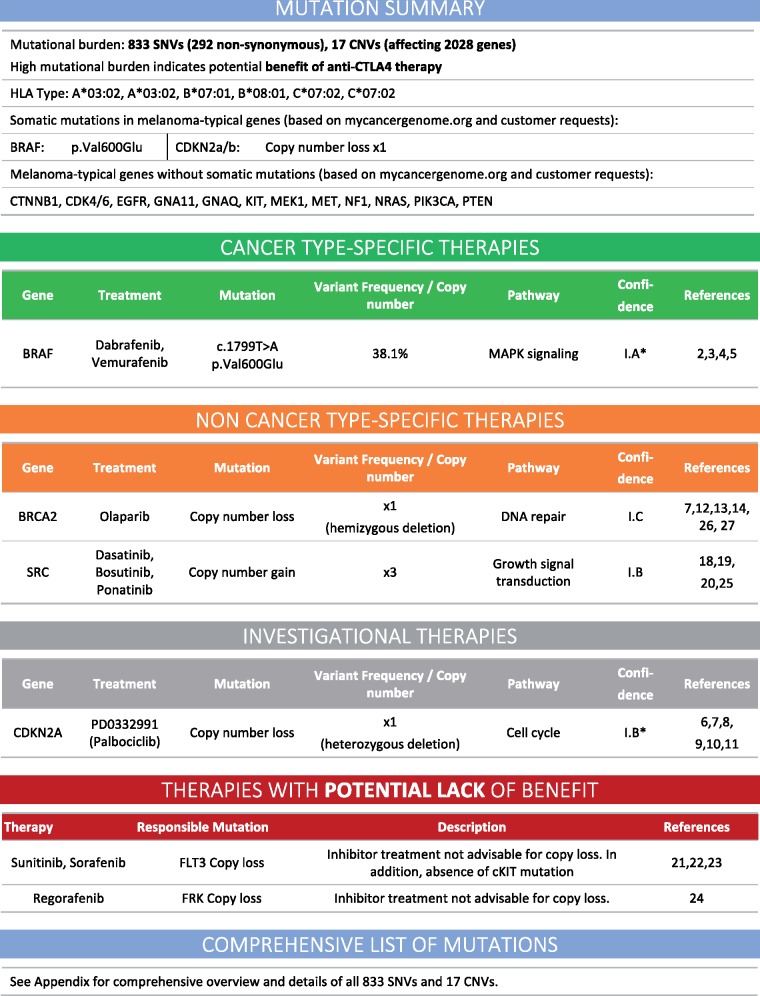
Example of concise report summary from an MTBZ report, including mutational burden, HLA-I type of the patient, mutational state of cancer-type-specific set of important genes, grouped according to level of approval.

We group therapies associated with detected somatic mutations into (i) cancer-type-specific therapies, (ii) non-cancer-type-specific therapies, (iii) investigational therapies and (iv) therapies potentially lacking benefit (Figure 3). The first category represents all suggested therapies which have been approved for the given cancer type by the local regulatory body, i.e. Swissmedic. The second group consists of therapies that are approved but not for the cancer type under consideration. This group is especially relevant owing to the increasing understanding that the genomic profile of a tumor is a better predictor for response than the tissue of origin alone [107]. By limiting this group to approved drugs only, it constitutes a source of available options to clinicians in Switzerland, where health insurances often approve the use of off-label treatments. The third group contains therapies which are not approved, but have been shown to be effective in preclinical studies and are currently in clinical trials, either open or ongoing. Although this group is usually based on low or insufficient levels of evidence, owing to singleton studies or only pre-clinical evidence, it frequently contains references to open clinical trials that the patient might be eligible for.

The final group includes therapies for which the genetic profile might cause reduced efficacy. In the fast-moving process of understanding the efficacy of novel therapeutics and their range of effects on different targets, a single trial showing lack of efficacy may be sufficient to exclude a therapy. For example, in a patient with neuroendocrine carcinoma, paclitaxel was a candidate drug for non-cancer-type-specific therapies. However, a clinical phase II study [108] showed that high-dose paclitaxel lacked antitumor activity and displayed significant hematologic toxicity in patients with advanced neuroendocrine tumors. Therefore, paclitaxel was listed as potentially lacking benefit.

In a first pilot study, we analyzed tumor biopsies and matched germ line samples from five metastatic melanoma patients with progressive disease on standard treatment and produced reports within a clinically relevant time period of 4-12 weeks from tumor biopsy.

Briefly, we performed WES and WGS on tumor biopsy samples together with a blood sample as matched normal control. Based on the pipeline framework described in [109], we use Trimmomatic [38] to remove adapters and quality trim the raw read sequences. We apply BWA [42] for read mapping and subsequently remove PCR duplicates using picard tools (http://broadinstitute.github.io/picard). Following the GATK best practices [46–48], we perform indel realignment and base recalibration previous to the variant calling. SNVs are called based on a combination of Mutect [61], Strelka [62] and VarScan2 [52] and further annotated based on various databases including dbSNP [54, 55], COSMIC [58] and ClinVar [57], and functional annotation based on dbNSFP [86, 110]. CNVs are called based on WGS, using BIC-seq2 [66]. All variants are compared against DGidb [93] to select the first set of candidates for possible targeted treatments based on reported drug–gene interactions. Candidate treatments are further prioritized, for instance based on the Swissmedic approval of the therapy, availability of clinical trials and treatment success in existing clinical studies. Finally, selected variants and respective treatment options are reported in the clinical report and discussed with the treating clinician.

In the five melanoma patients, we detected between 3 and 11 actionable aberrations per patient, most commonly in genes of the PI3K, cell cycle checkpoint and MAPK pathways. In two cases, the MTB recommended therapy based on our results: in one case, immunotherapy based on high mutational load, and in the other a chemotherapeutic drug based on a loss of a receptor activating the detoxification pathway of the drug. We observed a near-complete durable response in the first patient and a progression of disease in the second. The reasons for not following the report recommendations for the other patients were rapid decline of one patient’s condition and treatment with a newly approved immunotherapy regimen in two others.

Together with our clinical collaborators, we were able to draft a set of best practices on what to include in the report. These best practices are also viable for other disciplines outside of oncology. First of all, the report should begin with a concise summary of the most important findings. In our report, we focus on mutational load, the state of genes commonly mutated in the specific cancer type, a therapy summary and HLA-I type (Figure 3). Starting on page 2, the report should increase in depth such that the reader who would like to know more details can simply read on. Given the limited time to discuss a case in the MTB meeting, it is key that the most important facts can be grasped quickly from scanning the first page. Nevertheless, ideally, the report provides all information obtained from processing of the patient samples.

A selected list of clinical trial opportunities based on the molecular profile of the tumor are an important part of our report. Here, we refer to trials which are currently recruiting, thus offering a chance for the patient to get access to a potentially beneficial therapy, which might otherwise not be available. To allow the clinician to quickly assess the suitability of the trial, our report includes drug name, trial phase and title, as well as trial locations.

Given the rapid developments in molecular profiling technologies as well as in variant calling and annotation algorithms and databases, naturally, the MTBZ workflow is constant work in progress. In our most recent reports, for example, we started to incorporate transcriptomics data allowing us to detect up- and downregulation of genes and transcripts, gene fusions, alternative splicing events, as well as expression status of somatic mutations.

## Future directions

Bioinformatics workflows for the analysis and clinical interpretation of tumor molecular profiles have to various degrees been implemented in clinical trials and MTBs at specialized cancer centers and university hospitals worldwide. The initial results of these efforts are promising, but it has also become clear that exploiting the full potential of precision oncology faces many challenges.

One current bottleneck is efficient and precise annotation of variants. This step requires databases containing well-curated variants as well as their interactions with potential drugs. Text mining is a promising approach to accelerate and improve the process of not only curating variants across the globe, but also finding evidence in literature for interaction between drugs and genes as well as the effect of drug combinations [111]. Stronger proof for annotation in the form of globally curated variants and better literature evidence will ultimately speed up the process of interpreting results from molecular diagnostic testing, and thus overcome the bottleneck of precision oncology.

The rapid development of molecular profiling techniques will continue to provide new opportunities for precision oncology. For example, single-cell sequencing [112, 113], which allows for processing the DNA of hundreds and the gene expression levels of thousands of cells independently at the same time, will lead to increasing sensitivity levels with respect to mutation identification and the detection to tumor subclones, both of which are likely to affect treatment outcome. Further, multi-omics approaches will provide more insight into dysregulated pathways and increase the level of confidence in reporting an actionable variant when it can be confirmed by RNA, protein or epigenetic profiling. At the same time, multi-omics data will pose new bioinformatics challenges to integrate multiple data types and identify potentially efficacious treatments.

Moreover, powerful predictions of patient response to a personalized treatment strategy will come from functionally testing the suggested therapies on *ex vivo* tumor slices [114], in 2D or 3D cultures of the patient’s tumor or in patient-derived xenograft models [115]. This approach, although still in its infancy, will provide another level of therapeutic decision support for the MTB by allowing for the exclusion or confirmation of therapeutic efficacy and choice of the most efficacious drug combinations.

## Notes

Key Points

- Robust, reproducible, transparent and comprehensive bioinformatics pipelines are required for precision oncology, including molecular tumor boards and cancer basket trials.
- Variant calling, interpretation and annotation are at the core of improving cancer treatment by providing timely and reliable therapy recommendations.
- Clinical reporting of molecular findings is an important step that requires close interactions between bioinformaticians and clinicians.

## Notes

**Jochen Singer** is a PhD student in the Computational Biology Group at the Department of Biosystems Science and Engineering of ETH Zurich in Basel, Switzerland.

## Notes

**Anja Irmisch** is a cancer biology scientist in a translational oncology group at the Department of Dermatology at the University of Zurich Hospital in Zurich, Switzerland.

## Notes

**Hans-Joachim Ruscheweyh** is a postdoc in the Computational Biology Group at the Department of Biosystems Science and Engineering of ETH Zurich in Basel, Switzerland.

## Notes

**Franziska Singer** is a senior bioinformatics scientist in the Clinical Bioinformatics Unit of NEXUS Personalized Health Technologies at ETH Zurich in Basel, Switzerland.

## Notes

**Nora C. Toussaint** is a senior bioinformatics scientist in the Clinical Bioinformatics Unit of NEXUS Personalized Health Technologies at ETH Zurich in Zurich, Switzerland.

## Notes

**Mitchell P. Levesque** is associate professor for translational oncology at the Department of Dermatology at the University of Zurich Hospital in Zurich, Switzerland.

## Notes

**Daniel J. Stekhoven** is head of the Clinical Bioinformatics Unit of NEXUS Personalized Health Technologies at ETH Zurich in Zurich, Switzerland.

## Notes

**Niko Beerenwinkel** is associate professor of computational biology at the Department of Biosystems Science and Engineering of ETH Zurich in Basel, Switzerland.

## References

[1] Kotecha RS, Kees UR, Cole CH, et al. Rare childhood cancers–an increasing entity requiring the need for global consensus and collaboration. Cancer Med 2015;4(6):819–24. 25664881 10.1002/cam4.426 PMC4472204

[2] Meric-Bernstam F, Brusco L, Shaw K, et al. Feasibility of large-scale genomic testing to facilitate enrollment onto genomically matched clinical trials. J Clin Oncol 2015;33(25):2753–62. 26014291 10.1200/JCO.2014.60.4165 PMC4550690

[3] Burrell RA, McGranahan N, Bartek J, et al. The causes and consequences of genetic heterogeneity in cancer evolution. Nature 2013;501(7467):338–45. 24048066 10.1038/nature12625

[4] Gerlinger M, Rowan AJ, Horswell S, et al. Intratumor heterogeneity and branched evolution revealed by multiregion sequencing. N Engl J Med 2012;366(10):883–92. 22397650 10.1056/NEJMoa1113205 PMC4878653

[5] Oberg JA, Glade Bender JL, Sulis ML, et al. Implementation of next generation sequencing into pediatric hematology-oncology practice: moving beyond actionable alterations. Genome Med 2016;8:133. 28007021 10.1186/s13073-016-0389-6 PMC5180407

[6] Lane BR, Bissonnette J, Waldherr T, et al. Development of a center for personalized cancer care at a regional cancer center: feasibility trial of an institutional tumor sequencing advisory board. J Mol Diagn 2015;17(6):695–704. 26331835 10.1016/j.jmoldx.2015.07.003

[7] Pincez T, Clément N, Lapouble E, et al. Feasibility and clinical integration of molecular profiling for target identification in pediatric solid tumors. Pediatr Blood Cancer 2017;64(6):e26365. 10.1002/pbc.26365 27896933

[8] Bryce AH, Egan JB, Borad MJ, et al. Experience with precision genomics and tumor board, indicates frequent target identification, but barriers to delivery. Oncotarget 2017;8:27145–54. 28423702 10.18632/oncotarget.16057 PMC5432324

[9] Seeber A, Gastl G, Ensinger C, et al. Treatment of patients with refractory metastatic cancer according to molecular profiling on tumor tissue in the clinical routine: an interim-analysis of the ONCO-T-PROFILE project. Genes Cancer 2016;7(9-10):301–8. 28050231 10.18632/genesandcancer.121 PMC5115171

[10] Parker BA, Schwaederlé M, Scur MD, et al. Breast cancer experience of the molecular tumor board at the university of california, san diego moores cancer center. J Oncol Pract 2015;11(6):442–9. 26243651 10.1200/JOP.2015.004127

[11] Schwaederle M, Parker BA, Schwab RB, et al. Molecular tumor board: the University of California-San Diego Moores Cancer Center experience. Oncologist 2014;19(6):631–6. 24797821 10.1634/theoncologist.2013-0405 PMC4041669

[12] Beltran H, Eng K, Mosquera JM, et al. Whole-exome sequencing of metastatic cancer and biomarkers of treatment response. JAMA Oncol 2015;1(4):466–74. 26181256 10.1001/jamaoncol.2015.1313 PMC4505739

[13] Worst BC, van Tilburg CM, Balasubramanian GP, et al. Next-generation personalised medicine for high-risk paediatric cancer patients—the INFORM pilot study. Eur J Cancer 2016;65:91–101. 27479119 10.1016/j.ejca.2016.06.009

[14] Massard C, Michiels S, Ferté C, et al. High-throughput genomics and clinical outcome in hard-to-treat advanced cancers: results of the MOSCATO 01 trial. Cancer Discov 2017;7(6):586. 28365644 10.1158/2159-8290.CD-16-1396

[15] Conley BA, Gray R, Chen A, et al. Abstract CT101: NCI-molecular analysis for therapy choice (NCI-MATCH) clinical trial: interim analysis. Cancer Res 2016;76:CT101.

[16] Hamblin A, Wordsworth S, Fermont JM, et al. Clinical applicability and cost of a 46-gene panel for genomic analysis of solid tumours: retrospective validation and prospective audit in the UK National Health Service. PLoS Med 2017;14(2):e1002230. 28196074 10.1371/journal.pmed.1002230 PMC5308858

[17] Chapman PB, Hauschild A, Robert C, et al. Improved survival with vemurafenib in melanoma with BRAF V600E mutation. N Engl J Med 2011;364(26):2507–16. 21639808 10.1056/NEJMoa1103782 PMC3549296

[18] Hyman DM, Puzanov I, Subbiah V, et al. Vemurafenib in multiple nonmelanoma cancers with BRAF V600 mutations. N Engl J Med 2015;373(8):726–36. 26287849 10.1056/NEJMoa1502309 PMC4971773

[19] Jennings LJ, Arcila ME, Corless C, et al. Guidelines for validation of next-generation sequencing-based oncology panels: a joint consensus recommendation of the association for molecular pathology and college of american pathologists. J Mol Diagn 2017;19(3):341–65. 28341590 10.1016/j.jmoldx.2017.01.011 PMC6941185

[20] Aziz N, Zhao Q, Bry L, et al. College of American Pathologists’ laboratory standards for next-generation sequencing clinical tests. Arch Pathol Lab Med 2015;139(4):481–93. 25152313 10.5858/arpa.2014-0250-CP

[21] Matthijs G, Souche E, Alders M, et al. Guidelines for diagnostic next-generation sequencing. Eur J Hum Genet 2016;24(1):2–5. 26508566 10.1038/ejhg.2015.226 PMC4795226

[22] Guan P, Sung W-K. Structural variation detection using next-generation sequencing data: a comparative technical review. Methods 2016;102:36–49. 26845461 10.1016/j.ymeth.2016.01.020

[23] Alioto TS, Buchhalter I, Derdak S, et al. A comprehensive assessment of somatic mutation detection in cancer using whole-genome sequencing. Nat Commun 2015;6:10001. 26647970 10.1038/ncomms10001 PMC4682041

[24] Krøigård AB, Thomassen M, Lænkholm A-V, et al. Evaluation of nine somatic variant callers for detection of somatic mutations in exome and targeted deep sequencing data. PLoS One 2016;11(3):e0151664. 27002637 10.1371/journal.pone.0151664 PMC4803342

[25] Hofmann AL, Behr J, Singer J, et al. Detailed simulation of cancer exome sequencing data reveals differences and common limitations of variant callers. BMC Bioinformatics 2017;18:8. 28049408 10.1186/s12859-016-1417-7 PMC5209852

[26] Rigter T, Henneman L, Kristoffersson U, et al. Reflecting on earlier experiences with unsolicited findings: points to consider for next-generation sequencing and informed consent in diagnostics. Hum Mutat 2013;34(10):1322–8. 23784691 10.1002/humu.22370 PMC4285964

[27] Köster J, Rahmann S. Snakemake–a scalable bioinformatics workflow engine. Bioinformatics 2012;28(19):2520–2. 22908215 10.1093/bioinformatics/bts480

[28] Di Tommaso P, Chatzou M, Floden EW, et al. Nextflow enables reproducible computational workflows. Nat Biotechnol 2017;35(4):316–19. 28398311 10.1038/nbt.3820

[29] Vivian J, Rao AA, Nothaft FA, et al. Toil enables reproducible, open source, big biomedical data analyses. Nat Biotechnol 2017;35(4):314–16. 28398314 10.1038/nbt.3772 PMC5546205

[30] Sadedin SP, Pope B, Oshlack A. Bpipe: a tool for running and managing bioinformatics pipelines. Bioinformatics 2012;28(11):1525–6. 22500002 10.1093/bioinformatics/bts167

[31] Goecks J, Nekrutenko A, Taylor J, et al. Galaxy: a comprehensive approach for supporting accessible, reproducible, and transparent computational research in the life sciences. Genome Biol 2010;11(8):R86. 20738864 10.1186/gb-2010-11-8-r86 PMC2945788

[32] Pabinger S, Dander A, Fischer M, et al. A survey of tools for variant analysis of next-generation genome sequencing data. Brief Bioinformatics 2014;15(2):256–78. 23341494 10.1093/bib/bbs086 PMC3956068

[33] Cai L, Yuan W, Zhang Z, et al. In-depth comparison of somatic point mutation callers based on different tumor next-generation sequencing depth data. Sci Rep 2016;6:36540. 27874022 10.1038/srep36540 PMC5118795

[34] Nam J-Y, Kim NKD, Kim SC, et al. Evaluation of somatic copy number estimation tools for whole-exome sequencing data. Brief Bioinformatics 2016;17(2):185–92. 26210357 10.1093/bib/bbv055 PMC6283367

[35] Alkodsi A, Louhimo R, Hautaniemi S. Comparative analysis of methods for identifying somatic copy number alterations from deep sequencing data. Brief Bioinformatics 2015;16(2):242–54. 24599115 10.1093/bib/bbu004

[36] Del Fabbro C, Scalabrin S, Morgante M, et al. An extensive evaluation of read trimming effects on Illumina NGS data analysis. PLoS One 2013;8(12):e85024. 24376861 10.1371/journal.pone.0085024 PMC3871669

[37] Martin M. Cutadapt removes adapter sequences from high-throughput sequencing reads. EMBnet j 2011;17(1):10.

[38] Bolger AM, Lohse M, Usadel B. Trimmomatic: a flexible trimmer for Illumina sequence data. Bioinformatics 2014;30(15):2114–20. 24695404 10.1093/bioinformatics/btu170 PMC4103590

[39] Sturm M, Schroeder C, Bauer P. SeqPurge: highly-sensitive adapter trimming for paired-end NGS data. BMC Bioinformatics 2016;17:208. 27161244 10.1186/s12859-016-1069-7 PMC4862148

[40] Dodt M, Roehr JT, Ahmed R, et al. FLEXBAR-flexible barcode and adapter processing for next-generation sequencing platforms. Biology 2012;1(3):895–905. 24832523 10.3390/biology1030895 PMC4009805

[41] Fonseca NA, Rung J, Brazma A, et al. Tools for mapping high-throughput sequencing data. Bioinformatics 2012;28(24):3169–77. 23060614 10.1093/bioinformatics/bts605

[42] Li H, Durbin R. Fast and accurate short read alignment with Burrows-Wheeler transform. Bioinformatics 2009;25(14):1754–60. 19451168 10.1093/bioinformatics/btp324 PMC2705234

[43] Langmead B, Salzberg SL. Fast gapped-read alignment with Bowtie 2. Nat Methods 2012;9(4):357–9. 22388286 10.1038/nmeth.1923 PMC3322381

[44] Li H, Handsaker B, Wysoker A, et al. The Sequence Alignment/Map format and SAMtools. Bioinformatics 2009;25(16):2078–9. 19505943 10.1093/bioinformatics/btp352 PMC2723002

[45] Zhou W, Chen T, Zhao H, et al. Bias from removing read duplication in ultra-deep sequencing experiments. Bioinformatics 2014;30(8):1073–80. 24389657 10.1093/bioinformatics/btt771 PMC3982159

[46] McKenna A, Hanna M, Banks E, et al. The genome analysis toolkit: a mapreduce framework for analyzing next-generation DNA sequencing data. Genome Res 2010;20(9):1297–303. 20644199 10.1101/gr.107524.110 PMC2928508

[47] DePristo MA, Banks E, Poplin R, et al. A framework for variation discovery and genotyping using next-generation DNA sequencing data. Nat Genet 2011;43(5):491–8. 21478889 10.1038/ng.806 PMC3083463

[48] Van der Auwera GA, Carneiro MO, Hartl C, et al. From FastQ data to high confidence variant calls: the genome analysis toolkit best practices pipeline. Curr Protoc Bioinformatics 2013;11:11.10.1–33. 10.1002/0471250953.bi1110s43 PMC4243306 25431634

[49] Pirooznia M, Kramer M, Parla J, et al. Validation and assessment of variant calling pipelines for next-generation sequencing. Hum Genomics 2014;8:14. 25078893 10.1186/1479-7364-8-14 PMC4129436

[50] Tian S, Yan H, Kalmbach M, et al. Impact of post-alignment processing in variant discovery from whole exome data. BMC Bioinformatics 2016;17(1):403. 27716037 10.1186/s12859-016-1279-z PMC5048557

[51] Liu Q, Guo Y, Li J, et al. Steps to ensure accuracy in genotype and SNP calling from Illumina sequencing data. BMC Genomics 2012;13 (Suppl 8):S8. 10.1186/1471-2164-13-S8-S8 PMC3535703 23281772

[52] Koboldt DC, Zhang Q, Larson DE, et al. VarScan 2: somatic mutation and copy number alteration discovery in cancer by exome sequencing. Genome Res 2012;22(3):568–76. 22300766 10.1101/gr.129684.111 PMC3290792

[53] Kockan C, Hach F, Sarrafi I, et al. SiNVICT: ultra-sensitive detection of single nucleotide variants and indels in circulating tumour DNA. Bioinformatics 2017;33(1):26–34. 27531099 10.1093/bioinformatics/btw536

[54] Sherry ST, Ward M, Sirotkin K. dbSNP-database for single nucleotide polymorphisms and other classes of minor genetic variation. Genome Res 1999;9:677–9. 10447503

[55] Sherry ST, Ward MH, Kholodov M, et al. dbSNP: the NCBI database of genetic variation. Nucleic Acids Res 2001;29(1):308–11. 11125122 10.1093/nar/29.1.308 PMC29783

[56] Lek M, Karczewski KJ, Minikel EV, et al. Analysis of protein-coding genetic variation in 60, 706 humans. Nature 2016;536(7616):285–91. 27535533 10.1038/nature19057 PMC5018207

[57] Landrum MJ, Lee JM, Riley GR, et al. ClinVar: public archive of relationships among sequence variation and human phenotype. Nucleic Acids Res 2014;42:D980–5. 24234437 10.1093/nar/gkt1113 PMC3965032

[58] Forbes SA, Beare D, Gunasekaran P, et al. COSMIC: exploring the world’s knowledge of somatic mutations in human cancer. Nucleic Acids Res 2015;43:D805–11. 25355519 10.1093/nar/gku1075 PMC4383913

[59] Gerstung M, Beisel C, Rechsteiner M, et al. Reliable detection of subclonal single-nucleotide variants in tumour cell populations. Nat Commun 2012;3:811. 22549840 10.1038/ncomms1814

[60] Roth A, Ding J, Morin R, et al. JointSNVMix: a probabilistic model for accurate detection of somatic mutations in normal/tumour paired next-generation sequencing data. Bioinformatics 2012;28(7):907–13. 22285562 10.1093/bioinformatics/bts053 PMC3315723

[61] Cibulskis K, Lawrence MS, Carter SL, et al. Sensitive detection of somatic point mutations in impure and heterogeneous cancer samples. Nat Biotechnol 2013;31(3):213–19. 23396013 10.1038/nbt.2514 PMC3833702

[62] Saunders CT, Wong WSW, Swamy S, et al. Strelka: accurate somatic small-variant calling from sequenced tumor-normal sample pairs. Bioinformatics 2012;28(14):1811–17. 22581179 10.1093/bioinformatics/bts271

[63] Gerstung M, Papaemmanuil E, Campbell PJ. Subclonal variant calling with multiple samples and prior knowledge. Bioinformatics 2014;30(9):1198–204. 24443148 10.1093/bioinformatics/btt750 PMC3998123

[64] Zhao M, Wang Q, Wang Q, et al. Computational tools for copy number variation (CNV) detection using next-generation sequencing data: features and perspectives. BMC Bioinformatics 2013;14(Suppl 11):S1. 10.1186/1471-2105-14-S11-S1 PMC3846878 24564169

[65] Magi A, Tattini L, Cifola I, et al. EXCAVATOR: detecting copy number variants from whole-exome sequencing data. Genome Biol 2013;14(10):R120. 24172663 10.1186/gb-2013-14-10-r120 PMC4053953

[66] Xi R, Lee S, Xia Y, et al. Copy number analysis of whole-genome data using BIC-seq2 and its application to detection of cancer susceptibility variants. Nucleic Acids Res 2016;44(13):6274–86. 27260798 10.1093/nar/gkw491 PMC5772337

[67] Kuilman T, Velds A, Kemper K, et al. CopywriteR: DNA copy number detection from off-target sequence data. Genome Biol 2015;16:49. 25887352 10.1186/s13059-015-0617-1 PMC4396974

[68] Ye K, Schulz MH, Long Q, et al. Pindel: a pattern growth approach to detect break points of large deletions and medium sized insertions from paired-end short reads. Bioinformatics 2009;25(21):2865–71. 19561018 10.1093/bioinformatics/btp394 PMC2781750

[69] Zeitouni B, Boeva V, Janoueix-Lerosey I, et al. SVDetect: a tool to identify genomic structural variations from paired-end and mate-pair sequencing data. Bioinformatics 2010;26(15):1895–6. 20639544 10.1093/bioinformatics/btq293 PMC2905550

[70] Rausch T, Zichner T, Schlattl A, et al. DELLY: structural variant discovery by integrated paired-end and split-read analysis. Bioinformatics 2012;28(18):i333–9. 22962449 10.1093/bioinformatics/bts378 PMC3436805

[71] Layer RM, Chiang C, Quinlan AR, et al. LUMPY: a probabilistic framework for structural variant discovery. Genome Biol 2014;15(6):R84. 24970577 10.1186/gb-2014-15-6-r84 PMC4197822

[72] Tattini L, D’Aurizio R, Magi A. Detection of genomic structural variants from next-generation sequencing data. Front Bioeng Biotechnol 2015;3:92. 26161383 10.3389/fbioe.2015.00092 PMC4479793

[73] Ding L, Wendl MC, McMichael JF, et al. Expanding the computational toolbox for mining cancer genomes. Nat Rev Genet 2014;15(8):556–70. 25001846 10.1038/nrg3767 PMC4168012

[74] Dobin A, Davis CA, Schlesinger F, et al. STAR: ultrafast universal RNA-seq aligner. Bioinformatics 2013;29(1):15–21. 23104886 10.1093/bioinformatics/bts635 PMC3530905

[75] Kim D, Pertea G, Trapnell C, et al. TopHat2: accurate alignment of transcriptomes in the presence of insertions, deletions and gene fusions. Genome Biol 2013;14(4):R36. 23618408 10.1186/gb-2013-14-4-r36 PMC4053844

[76] Anders S, Pyl PT, Huber W. HTSeq–a Python framework to work with high-throughput sequencing data. Bioinformatics 2015;31(2):166–9. 25260700 10.1093/bioinformatics/btu638 PMC4287950

[77] Liao Y, Smyth GK, Shi W. FeatureCounts: an efficient general purpose program for assigning sequence reads to genomic features. Bioinformatics 2014;30(7):923–30. 24227677 10.1093/bioinformatics/btt656

[78] Love MI, Huber W, Anders S. Moderated estimation of fold change and dispersion for RNA-seq data with DESeq2. Genome Biol 2014;15(12):550. 25516281 10.1186/s13059-014-0550-8 PMC4302049

[79] McCarthy DJ, Chen Y, Smyth GK. Differential expression analysis of multifactor RNA-Seq experiments with respect to biological variation. Nucleic Acids Res 2012;40(10):4288–97. 22287627 10.1093/nar/gks042 PMC3378882

[80] Robinson MD, McCarthy DJ, Smyth GK. edgeR: a Bioconductor package for differential expression analysis of digital gene expression data. Bioinformatics 2010;26(1):139–40. 19910308 10.1093/bioinformatics/btp616 PMC2796818

[81] Carithers LJ, Moore HM. The genotype-tissue expression (GTEx) project. Biopreserv Biobank 2015;13(5):307–8. 26484569 10.1089/bio.2015.29031.hmm PMC4692118

[82] Leek JT, Johnson WE, Parker HS, et al. The sva package for removing batch effects and other unwanted variation in high-throughput experiments. Bioinformatics 2012;28(6):882–3. 22257669 10.1093/bioinformatics/bts034 PMC3307112

[83] Griffith M, Spies NC, Krysiak K, et al. CIViC is a community knowledgebase for expert crowdsourcing the clinical interpretation of variants in cancer. Nat Genet 2017;49(2):170–4. 28138153 10.1038/ng.3774 PMC5367263

[84] Cingolani P, Platts A, Wang LL, et al. A program for annotating and predicting the effects of single nucleotide polymorphisms, SnpEff: SNPs in the genome of Drosophila melanogaster strain w1118; iso-2; iso-3. Fly 2012;6(2):80–92. 22728672 10.4161/fly.19695 PMC3679285

[85] Wang K, Li M, Hakonarson H. ANNOVAR: functional annotation of genetic variants from high-throughput sequencing data. Nucleic Acids Res 2010;38(16):e164. 20601685 10.1093/nar/gkq603 PMC2938201

[86] Liu X, Wu C, Li C, et al. dbNSFP v3.0: a one-stop database of functional predictions and annotations for human nonsynonymous and splice-site SNVs. Hum Mutat 2016;37(3):235–41. 26555599 10.1002/humu.22932 PMC4752381

[87] Choi Y, Sims GE, Murphy S, et al. Predicting the functional effect of amino acid substitutions and indels. PLoS One 2012;7(10):e46688. 23056405 10.1371/journal.pone.0046688 PMC3466303

[88] Vogelstein B, Papadopoulos N, Velculescu VE, et al. Cancer genome landscapes. Science 2013;339(6127):1546–58. 23539594 10.1126/science.1235122 PMC3749880

[89] Tamborero D, Gonzalez-Perez A, Perez-Llamas C, et al. Comprehensive identification of mutational cancer driver genes across 12 tumor types. Sci Rep 2013;3:2650. 24084849 10.1038/srep02650 PMC3788361

[90] The UniProt Consortium. UniProt: the universal protein knowledgebase. Nucleic Acids Res 2017;45:D158–69. 27899622 10.1093/nar/gkw1099 PMC5210571

[91] Rubio-Perez C, Tamborero D, Schroeder MP, et al. In silico prescription of anticancer drugs to cohorts of 28 tumor types reveals targeting opportunities. Cancer Cell 2015;27(3):382–96. 25759023 10.1016/j.ccell.2015.02.007

[92] Gonzalez-Perez A, Perez-Llamas C, Deu-Pons J, et al. IntOGen-mutations identifies cancer drivers across tumor types. Nat Methods 2013;10(11):1081–2. 24037244 10.1038/nmeth.2642 PMC5758042

[93] Wagner AH, Coffman AC, Ainscough BJ, et al. DGIdb 2.0: mining clinically relevant drug-gene interactions. Nucleic Acids Res 2016;44:D1036–44. 26531824 10.1093/nar/gkv1165 PMC4702839

[94] Thurnherr T, Singer F, Stekhoven DJ, et al. Genomic variant annotation workflow for clinical applications. F1000Research 2016;5:1963. 27990260 10.12688/f1000research.9357.1 PMC5130070

[95] Chakravarty D, Gao J, Phillips S, et al. OncoKB: a precision oncology knowledge base. JCO Precision Oncology 2017. doi: 10.1200/PO.17.00011. 10.1200/PO.17.00011 PMC5586540 28890946

[96] Schneider L, Stöckel D, Kehl T, et al. DrugTargetInspector: an assistance tool for patient treatment stratification. Int J Cancer 2016;138(7):1765–76. 26501925 10.1002/ijc.29897

[97] Good BM, Ainscough BJ, McMichael JF, et al. Organizing knowledge to enable personalization of medicine in cancer. Genome Biol 2014;15:438. 25222080 10.1186/s13059-014-0438-7 PMC4281950

[98] Le Tourneau C, Kamal M, Tsimberidou A-M, et al. Treatment algorithms based on tumor molecular profiling: the essence of precision medicine trials. J Natl Cancer Inst 2016;108(4):djv362. 10.1093/jnci/djv362 PMC4830395 26598514

[99] Li MM, Datto M, Duncavage EJ, et al. Standards and guidelines for the interpretation and reporting of sequence variants in cancer: a joint consensus recommendation of the association for molecular pathology, american society of clinical oncology, and college of american pathologists. J Mol Diagn 2017;19(1):4–23. 27993330 10.1016/j.jmoldx.2016.10.002 PMC5707196

[100] Whirl-Carrillo M, McDonagh EM, Hebert JM, et al. Pharmacogenomics knowledge for personalized medicine. Clin Pharmacol Ther 2012;92(4):414–17. 22992668 10.1038/clpt.2012.96 PMC3660037

[101] Singer F, Irmisch A, Toussaint NC, et al. Establishing molecular diagnostics in Swiss clinics. 2017, In preparation. 10.1186/s12911-018-0680-0 PMC6206832 30373609

[102] Snyder A, Makarov V, Merghoub T, et al. Genetic basis for clinical response to CTLA-4 blockade in melanoma. N Engl J Med 2014;371(23):2189–99. 25409260 10.1056/NEJMoa1406498 PMC4315319

[103] Alexandrov LB, Nik-Zainal S, Wedge DC, et al. Signatures of mutational processes in human cancer. Nature 2013;500(7463):415–21. 23945592 10.1038/nature12477 PMC3776390

[104] Szolek A, Schubert B, Mohr C, et al. OptiType: precision HLA typing from next-generation sequencing data. Bioinformatics 2014;30(23):3310–16. 25143287 10.1093/bioinformatics/btu548 PMC4441069

[105] Legat A, Maby-El Hajjami H, Baumgaertner P, et al. Vaccination with LAG-3Ig (IMP321) and peptides induces specific CD4 and CD8 T-cell responses in metastatic melanoma patients–report of a phase I/IIa clinical trial. Clin Cancer Res 2016;22(6):1330–40. 26500235 10.1158/1078-0432.CCR-15-1212

[106] Cerami E, Gao J, Dogrusoz U, et al. The cBio cancer genomics portal: an open platform for exploring multidimensional cancer genomics data. Cancer Discov 2012;2(5):401–4. 22588877 10.1158/2159-8290.CD-12-0095 PMC3956037

[107] Redig AJ, Jänne PA. Basket trials and the evolution of clinical trial design in an era of genomic medicine. J Clin Oncol 2015;33(9):975–7. 25667288 10.1200/JCO.2014.59.8433

[108] Ansell SM, Pitot HC, Burch PA, et al. A phase II study of high-dose paclitaxel in patients with advanced neuroendocrine tumors. Cancer 2001;91(8):1543–8. 11301403 10.1002/1097-0142(20010415)91:8<1543::aid-cncr1163>3.0.co;2-n

[109] Singer J, Ruscheweyh H-J, Hofmann AL, et al. NGS-pipe: a flexible, easily extendable, and highly configurable framework for NGS analysis. Bioinformatics 2017. doi: 10.1093/bioinformatics/btx540. 10.1093/bioinformatics/btx540 PMC5870795 28968639

[110] Liu X, Jian X, Boerwinkle E. dbNSFP: a lightweight database of human nonsynonymous SNPs and their functional predictions. Hum Mutat 2011;32(8):894–9. 21520341 10.1002/humu.21517 PMC3145015

[111] Singhal A, Simmons M, Lu Z, et al. Text mining genotype-phenotype relationships from biomedical literature for database curation and precision medicine. PLoS Comput Biol 2016;12(11):e1005017. 27902695 10.1371/journal.pcbi.1005017 PMC5130168

[112] Gawad C, Koh W, Quake SR. Single-cell genome sequencing: current state of the science. Nat Rev Genet 2016;17(3):175–88. 26806412 10.1038/nrg.2015.16

[113] Svensson V, Natarajan KN, Ly L-H, et al. Power analysis of single-cell RNA-sequencing experiments. Nat Methods 2017;14(4):381–7. 28263961 10.1038/nmeth.4220 PMC5376499

[114] Davies EJ, Dong M, Gutekunst M, et al. Capturing complex tumour biology *in vitro*: histological and molecular characterisation of precision cut slices. Sci Rep 2015;5:17187. 26647838 10.1038/srep17187 PMC4673528

[115] Pauli C, Hopkins BD, Prandi D, et al. Personalized *in vitro* and *in vivo* cancer models to guide precision medicine. Cancer Discov 2017;7(5):462–77. 28331002 10.1158/2159-8290.CD-16-1154 PMC5413423

